# Muscle activation strategies of people with early-stage Parkinson’s during walking

**DOI:** 10.1186/s12984-021-00932-1

**Published:** 2021-09-08

**Authors:** Sana M. Keloth, Sridhar P. Arjunan, Sanjay Raghav, Dinesh Kant Kumar

**Affiliations:** 1 Biosignals Lab, School of Engineering, RMIT University, Melbourne, VIC Australia; 2 Department of Electronics and Instrumentation, SRM Institute of Science and Technology, Chennai, India; 3 Monash Health, Clayton, VIC Australia

**Keywords:** Parkinson’s disease, Wearable sensors, Muscle activation, Gait analysis

## Abstract

**Introduction:**

Some people with Parkinson’s disease (PD) frequently have an unsteady gait with shuffling, reduced strength, and increased rigidity. This study has investigated the difference in the neuromuscular strategies of people with early-stage PD, healthy older adults (HOA) and healthy young adult (HYA) during short-distance walking.

**Method:**

Surface electromyogram (sEMG) was recorded from tibialis anterior (TA) and medial gastrocnemius (MG) muscles along with the acceleration data from the lower leg from 72 subjects—24 people with early-stage PD, 24 HOA and 24 HYA during short-distance walking on a level surface using wearable sensors.

**Results:**

There was a significant increase in the co-activation, a reduction in the TA modulation and an increase in the TA-MG lateral asymmetry among the people with PD during a level, straight-line walking. For people with PD, the gait impairment scale was low with an average postural instability and gait disturbance (PIGD) score = 5.29 out of a maximum score of 20. Investigating the single and double support phases of the gait revealed that while the muscle activity and co-activation index (CI) of controls modulated over the gait cycle, this was highly diminished for people with PD. The biggest difference between CI of controls and people with PD was during the double support phase of gait.

**Discussion:**

The study has shown that people with early-stage PD have high asymmetry, reduced modulation, and higher co-activation. They have reduced muscle activity, ability to inhibit antagonist, and modulate their muscle activities. This has the potential for diagnosis and regular assessment of people with PD to detect gait impairments using wearable sensors.

## Background

Parkinson’s disease (PD) is a progressive neurodegenerative disorder with gait impairment and posture dysfunction being common symptoms [1]. Reduced production of dopamine neurotransmitter in the substantia nigra leads to the excessive inhibition of the basal ganglia loop which causes loss of habitual patterns associated with walking [2] and decreased range of limb movement [3]. Most of the people with PD are high-risk fallers, and often have an unsteady gait with shuffling, reduced strength, and increased rigidity [4]. Often there is a reduced pre-swing phase which is caused by decreased plantar forces at the forefoot, resulting in reduced leg acceleration during swing phase, stride length and gait speed [5–7]. However, human gait is a result of a number of non-neurological factors such as skeletal deformities and it is possible to miss some of the gait impairment symptoms in the early-stage of the disease.

The cycle of the repeatable movements during the gait is defined as the gait cycle. The gait interval, also known as stride interval [8, 9], is the time between subsequent heel strikes of the same foot. It consists of two main sub-phases: stance and swing. The stance phase begins with the heel strike and ends with toe-off of the same foot [10]. There are two double support, and one single-limb support parts of the stance phase [11, 12], followed by the swing phase, when the foot is unsupported after the toe-off [13].

Surface electromyography (sEMG) of gait has applications for diagnosis of conditions such as abnormal loading response [14, 15] which in people with PD can lead to postural instability [16]. It has also been reported that they have lower gastrocnemius muscle activity during the stance phase [17], reduced ability to modulate their activation pattern [18] and their activity is not steady [19]. People with PD also have reduced tibialis anterior (TA) during the stance phase [17] and reduced TA amplitude during late swing [20] which reflects the impairment in motor control with limited control of foot and stride length [21].

Lang et al. [22] reported that people with PD have higher co-activation of agonist-antagonist muscles around the ankle. Co-activation stabilizes the joints [23], but excessive co-activation produces negative work, reduced net torque and increased rigidity [24]. Ervilha et al. [25] reported that the Co-activation Index (CI) is obtained from the sEMG of the opposing muscles. While CI increases with age and disease, in older adults it is associated with an increase in the muscle activation during mid-stance [26], but patients with PD have reduction in the overall muscle activity. There is also a significant change to CI over the different sub-phases of the gait for older adults when compared to young [26] but this has not been studied for people with PD.

People have a natural tendency to use one side of the body in a voluntary motor task and is called lateral preference [27]. However, the gait of most people is generally symmetrical. Patients with PD have higher gait asymmetry [28, 29], but the associated changes in the muscle activation strategy of the right and left leg muscles was not found [30, 31]. One potential error in this could be that the dominant side could have changed caused by the onset of the disease [32]. To overcome this, Asymmetry Index (AI) was introduced, where the ‘higher’ vs ‘lower’ sides were compared and significant gait asymmetry was observed [19]. However, asymmetry differences between different phases of the gait cycle has not been reported.

During walking and maintained posture, there is a cyclic variation of the muscle activity of TA and MG muscles in healthy people having high sEMG modulation index (MI) [33]. This conserves energy and provides stability [19, 33]. A greater MI indicates a larger number of motor units recruited and de-recruited and has a bigger range of activity [34]. People with PD have reduced modulation while maintaining balance [22] but their MI during gait has not been reported.

The decline of Postural Instability and Gait Disturbance (PIGD), a sub score of the UPDRS-III is one of the important parameters for monitoring the progression of the disease. The aim of this experimental study was to identify the neuromuscular differences between people with PD with early-stage PIGD and controls during walking using wearable sensors and suitable for a typical clinic. We hypothesized that PD would exhibit asymmetrical muscle activity, reduced modulation, and increased concurrent activation of muscles during regular walking. This study has the potential for monitoring people with PD for early detection of gait impairment before kinematic and clinical changes in gait are detectable.

## Materials and methods

### Participants

The study based on a statistical power of 80%, recruited 72 participants: 24 people with early-stage PD, 24 healthy older adults (HOA) and 24 healthy young adult (HYA). All people with PD were from the outpatient clinic at Dandenong Neurology, Melbourne, Australia; HOA approximately matched the age and gender of the people with PD and were from independent living aged-care facilities and HYA were from RMIT University in the age 18–30 years. Patients with PD were in stage 1 of the disease. All participants reported themselves to be lower-limb right side dominant through the questionnaire. The demographic details are provided in Table 1.

**Table 1:** The demographic details in mean (± SD) of three group—PD, HOA, HYA participants

	PD	HOA	HYA	*p*-value
Demographic variables				PD and HOA
Age (Years)	71.91 ± 8.64	67.25 ± 3.77	27.91 ± 2.43	0.09
Gender (male/female)	17/7	17/7	18/6	1
Height (cm)	169.26 ± 8.89	166.54 ± 8.20	161.33 ± 4.26	0.13
Weight (kg)	81.25 ± 15.86	73.58 ± 12.46	60.29 ± 8.07	0.09
Body mass index (BMI) (kg/m^2^)	28.47 ± 5.85	26.64 ± 5.06	23.16 ± 3.06	0.25

Differences in age, height, weight and BMI were compared across the PD and HOA groups using Mann–Whitney U tests. Gender difference between PD and HOA was performed using chi-squared test

The exclusion criteria for people with PD were clinically observed or self-reported skeletal injuries, neurological or muscular-skeletal diseases other than PD, and Movement Disorder Society Unified Parkinson’s Disease Rating Scale (MDS-UPDRS) > 50. People with PD were of mixed phenotypes, and all were in their ON phase of the medication cycle. HOA and HYA groups were chosen such that the gender ratio (male: female) was like that of the PD group.

Participant’s demographic data, medical history, psychiatric history, current medication, and PD history (duration, symptoms, previous medication time, progression) were collected. The severity of the motor symptoms of PD were assessed according to the guidelines of the motor examination section of the MDS-UPDRS, Hoehn and Yahr (H & Y) scale by the examiners who were trained and supervised by Movement Disorder specialist. UPDRS III PIGD is the sum of sub-score comprising of arising from a chair (Item 3.9), posture (Item 3.13), gait (Item 3.10), postural stability (Item 3.12) and bradykinesia (Item 3.14) [35]. The experiments were conducted in accordance with the Helsinki declaration on human experiments (revised 2006), and the protocol was approved by RMIT University Human Research Ethics Committee (BSEHAPP 22-15). The experiment was explained to the participants and their written informed consent was obtained before the experiment. Table 1 shows the demographic details and Table 2 shows the clinical details of the three groups. The average PIGD of people with PD was 5.29 and H & Y of 2.27, which indicates that these patients were in their early-stage of disease, without balance impairment.

**Table 2:** The clinical characteristics in mean (± SD) of people with PD participants

Clinical variables	PD
Disease duration (years)	4.27 ± 3.15
Time since last medication (hours)	3.67 ± 1.68
UPDRS III	25.69 ± 10.95
UPDRS PIGD subscore	5.29 ± 3.07
H &Y scale	2.27 ± 0.94
Levodopa dosage mg/day	456.72 ± 148.23
Range of H &Y scale	1–3
Tremor at rest (lower limb)	0.125 ± 0.33
UPDRS rigidity (Item 3.3)	1.16 ± 0.83
UPDRS leg agility (Item 3.8)	1.27 ± 0.19
UPDRS gait (Item 3.10)	1.08 ± 0.77
UPDRS postural stability (Item 3.12)	1.41 ± 0.71
UPDRS body bradykinesia (Item 3.14)	1.04 ± 0.75

### Data recording

The effectiveness of using inertial movement sensors for detecting gait events has been shown [36] and were used to detect the sub-phases of the gait. A wireless Trigno (Delsys, Boston, USA) system has one channel each for acceleration, rotation and magnetic field and one channel active electrode for surface electromyogram (sEMG) with an inter-electrode distance of 20 mm, and bandwidth of 20–450 Hz. The maximum wireless operating range of the sensor is 20 m. The sampling rate of the sEMG signals is 2000 samples/second, of the accelerometer and gyroscope signals is 148 samples/second and of the magnetometer signals is 74 samples/second.

The electrodes were placed on the medial gastrocnemii muscle (MG) and TA muscles of the left and right legs, and the positioning was based on the SENIAM recommendation. The accelerometer that was embedded in the sensor placed in the TA muscle was used to compute the gait intervals and studying the gait events which has been described in our earlier work [37].

### Experiment protocol

The protocol required the participants to walk along a path marked on a level floor with white markers in a clinic. To reduce confounding factors due to turning, only the last segment with straight-line walking was considered in this study. The length of the straight-line walking segments was greater than 2 m and all participants had not less than 2 full bipedal gait cycles. The participants walked at their own convenient pace. Assessments were also video recorded for reference and second opinion. A detailed description of the experiment protocol has been reported in our earlier paper [38] in which the acceleration data was analyzed to study the gait parameters for different type of walking.

### Pre-processing of the signal

Two full gait cycles were studied for each participant. The recordings were pre-processed to remove noise. The sEMG was filtered using 20–450 Hz, sixth-order Butterworth band-pass filter. The envelope was obtained using a root mean square (RMS) moving average with a 100 ms window and an overlapping of 10 ms. The noise in the accelerometer and gyrometer was reduced using a second-order bandpass Butterworth filter with cut-off frequency 0.01–20 Hz.

### Gait cycle identification

The gait cycle of each participant was calculated from the acceleration and angular velocity curve obtained from the sensor. The heel strike of the leg was identified as the highest peak in the acceleration curve. The difference between the two-consecutive heel strikes of the same leg was taken as a gait cycle. A detailed description of the method has been reported in our earlier paper [38].

### Normalization of sEMG features

sEMG amplitude, frequency and duration are affected by many factors such as electrode placements, subcutaneous fat thickness, muscle fibre type and speed of the actions. To reduce the inter-subject and inter-experiment differences, amplitude normalization was done based on the peak root-mean-square (RMS) during the gait cycle for each individual and each muscle separately [39]. The data was then normalized in the time domain such that the complete gait-cycle corresponded to 100 data points [39].

### Gait and sEMG feature extraction

The computation of the gait sub-phase parameters has been explained in detail [38]. Three sEMG features were calculated—CI, MI and AI, and these have been described below.

#### Co-activation index (CI)

CI is the measure of co-activation, and in this study, computed from the normalized sEMG of both TA and MG muscle. A larger value of CI denotes the simultaneous activation of TA-MG muscles around the joint, which can result in altered mechanical properties of the limb [40]. A smaller value of CI corresponds to alternate activation of TA and MG muscle and is referred to as reciprocal inhibition [41].

The TA-MG CI was calculated by dividing the area of TA-MG overlap by the total area of TA-MG muscle as given in expression (1) [42].

1 $${\text{CI}} = \frac{{Overlapping\;area\;of\;TA\;and\;MG\;muscle}}{{Area\;of\;TA\;muscle + area\;of\;MG\;muscle}}$$

CI was calculated for total gait cycle (0–100%), and the sub-phases—1DS, SS, 2DS and SW.

#### Modulation index (MI)

MI is the measure of the range of muscle activation and was calculated from expression (2)

2 $$MI=\frac{{EMG}_{max}-{EMG}_{min}}{{EMG}_{max}}*100$$

where EMG_max_ is the maximum and EMG_min_ is the minimum RMS of sEMG activity. Linear envelope was obtained using a root mean square (RMS) moving average with a 100 ms window and an overlapping of 10 ms. A larger value of MI denotes that the muscles produce a phasic burst of activity followed by relaxation and has a bigger range of activity during the movement. Smaller MI indicates that muscle activity did not vary significantly over the cycle [34].

#### Asymmetry index (AI)

Early-stage people with PD exhibit bilateral asymmetry during walking. The tendency of the person to use one side of the body in a voluntary motor task is called lateral preference [27]. Bilateral muscle asymmetry was calculated using AI as the absolute value from expression (3) and as described by Bailey et al. [19]. After computing the EMG-RMS of TA and MG muscles for both legs, the AI was calculated using expression (3), where leg 1 corresponds to the higher value of sEMG RMS and leg 2 corresponds to the lower value of sEMG RMS for each sub-phases of gait.

3 $$AI = 100 - \left( {\frac{{leg1}}{{leg2}}*100} \right)$$

### Statistical analysis

The Shapiro–Wilk Test was performed for the demographic variables—age, height, and weight and data was not found to be normally distributed (*p* < 0.02). Chi-squared test was performed for finding the gender difference between PD and HOA. Differences in age, height, and weight were compared across the PD and HOA groups, and height and weight between HOA and HYA using Mann–Whitney U tests after testing the normal distribution using Shapiro Wilk Test. No statistically significant differences were found for either of the 2 tests. Kruskal–Wallis test without Type I correction was used to check for the difference between PD, HOA, and HYA based on CI, AI and MI obtained from sEMG [43]. Bonferroni post-hoc test was then performed to identify the difference between the three groups. Spearman correlation was performed to study the relation between sEMG features and clinical parameters. The patients were grouped into two groups based on the H & Y stages: PD 1 with H & Y between 1 to 2 and PD2 being patients with H & Y in the range between 2.5 and 3. Out of 24 patients with PD, 15 were classified as PD1 and 9 as PD2. The criteria for correlation used were—weak (values of 0.25–0.50), moderate (values of 0.50–0.75), and strong (values of 0.75 and above) [44].

## Results

There was no statistically significant difference for each demographic variable: age (U = 169, *p* = 0.09), height (U = 176, *p* = 0.13), weight (U = 162, *p* = 0.09) tested using Mann–Whitney U test. There was a small, and insignificant difference of age and weight between HOA and people with PD. Being statistically not significant, these differences were within the acceptable range for the experiments.

**Fig. 1:**
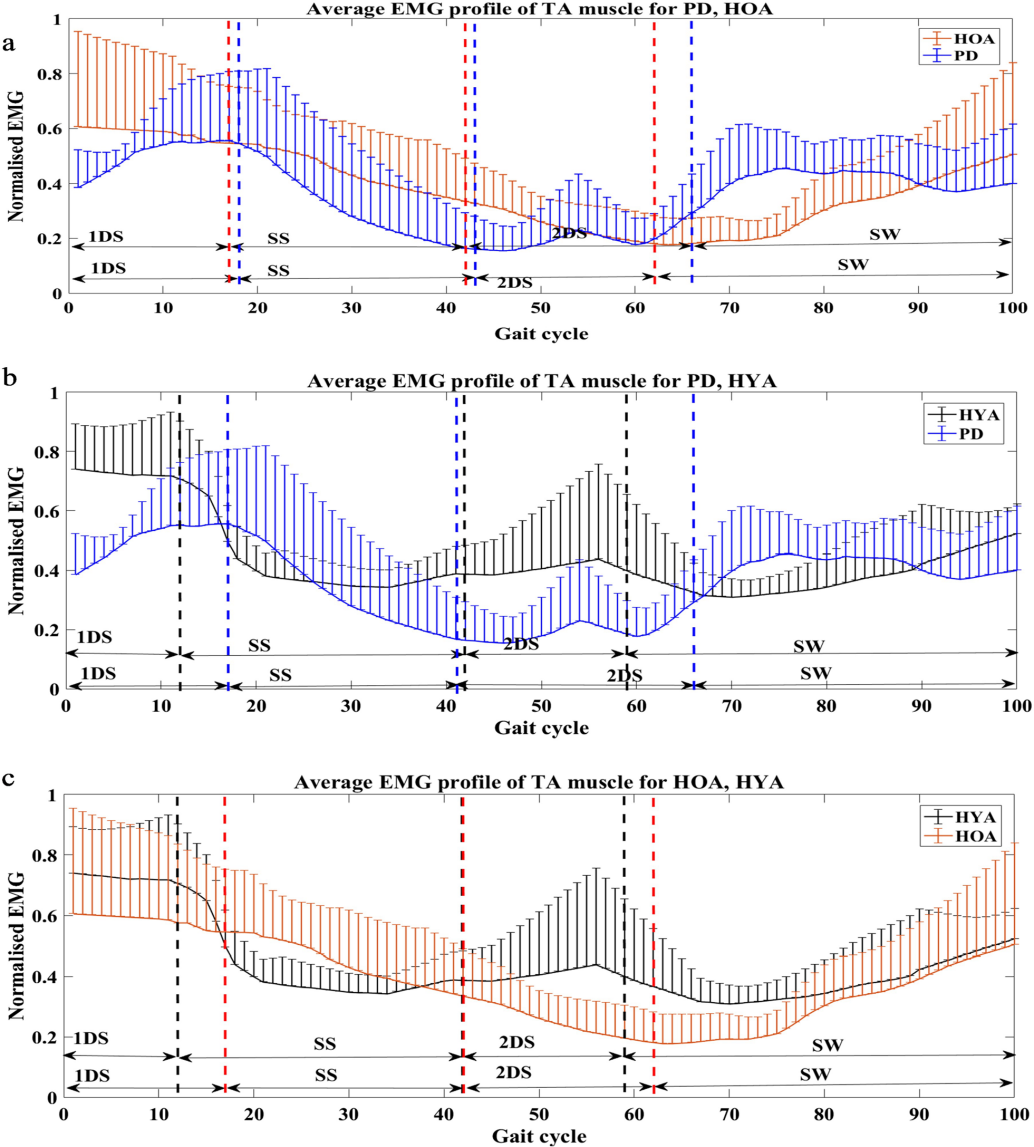
Plot showing the average sEMG profiles with the standard deviation bars of TA muscle between **a** PD and HOA subjects, **b** PD and HYA subjects, **c** HOA and HYA subjects for sub-phases of gait cycle (first double support (1DS), single support (SS), second double support phase (2DS) and swing (SW) phase)

Figure 1 shows the sEMG profile of TA muscle for people with PD, HOA and HYA groups. Visual observation showed that TA muscle is more active in people with PD during the SW phase of gait when compared to age matched controls. TA was also found to be more active among the HYA for all phases except the SS phase of gait.

**Fig. 2:**
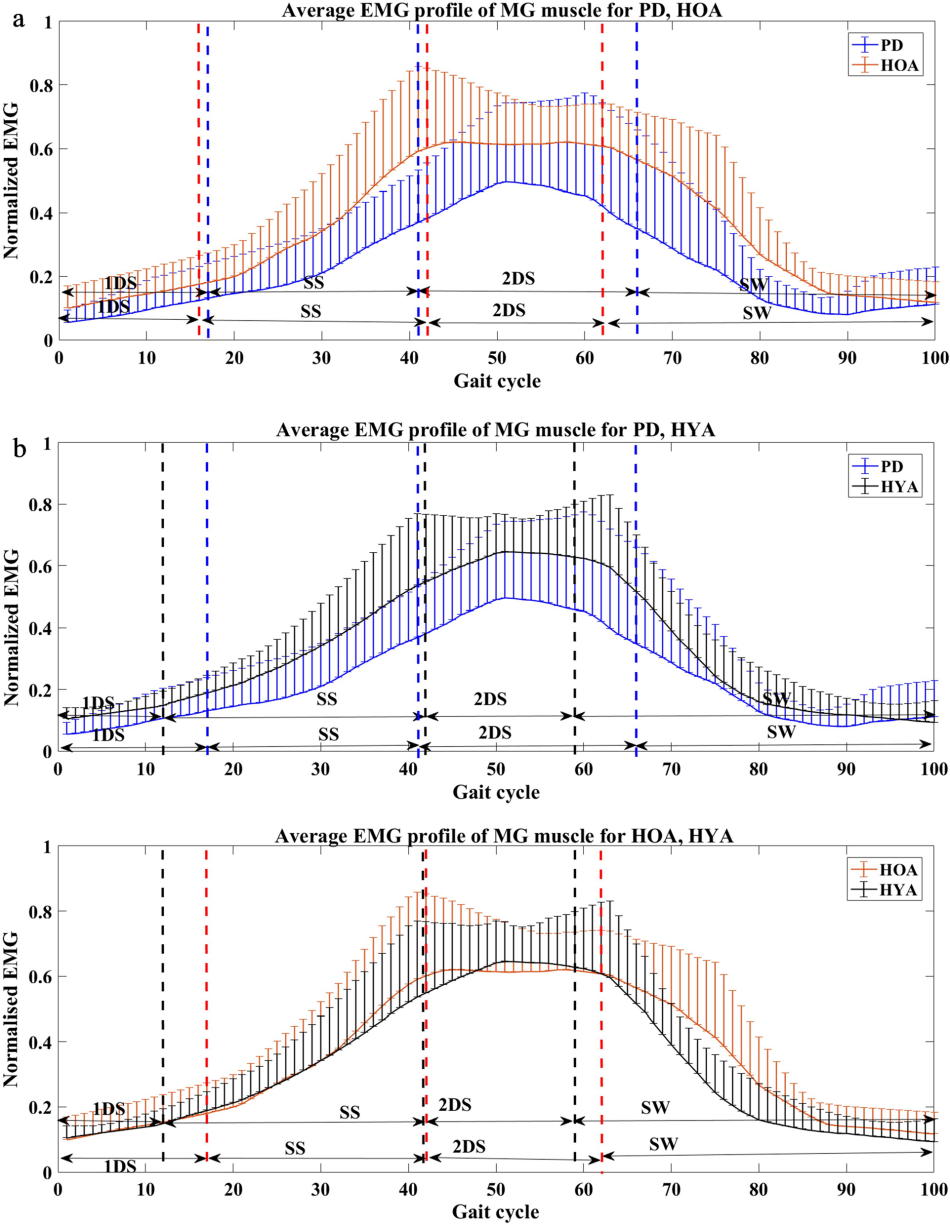
Plot showing the average sEMG profiles with the standard deviation bars of MG muscle between **a** PD and HOA subjects, **b** PD and HYA subjects, **c** HOA and HYA subjects for sub-phases of gait cycle (first double support (1DS), single support (SS), second double support phase (2DS) and swing (SW) phase)

Figure 2 shows the sEMG profile of MG muscle for PD, HOA and HYA groups. It was visually observed that for people with PD, the MG muscle is less active compared to the other groups during all phase of gait.

### Co-activation of TA and MG muscle

**Fig. 3:**
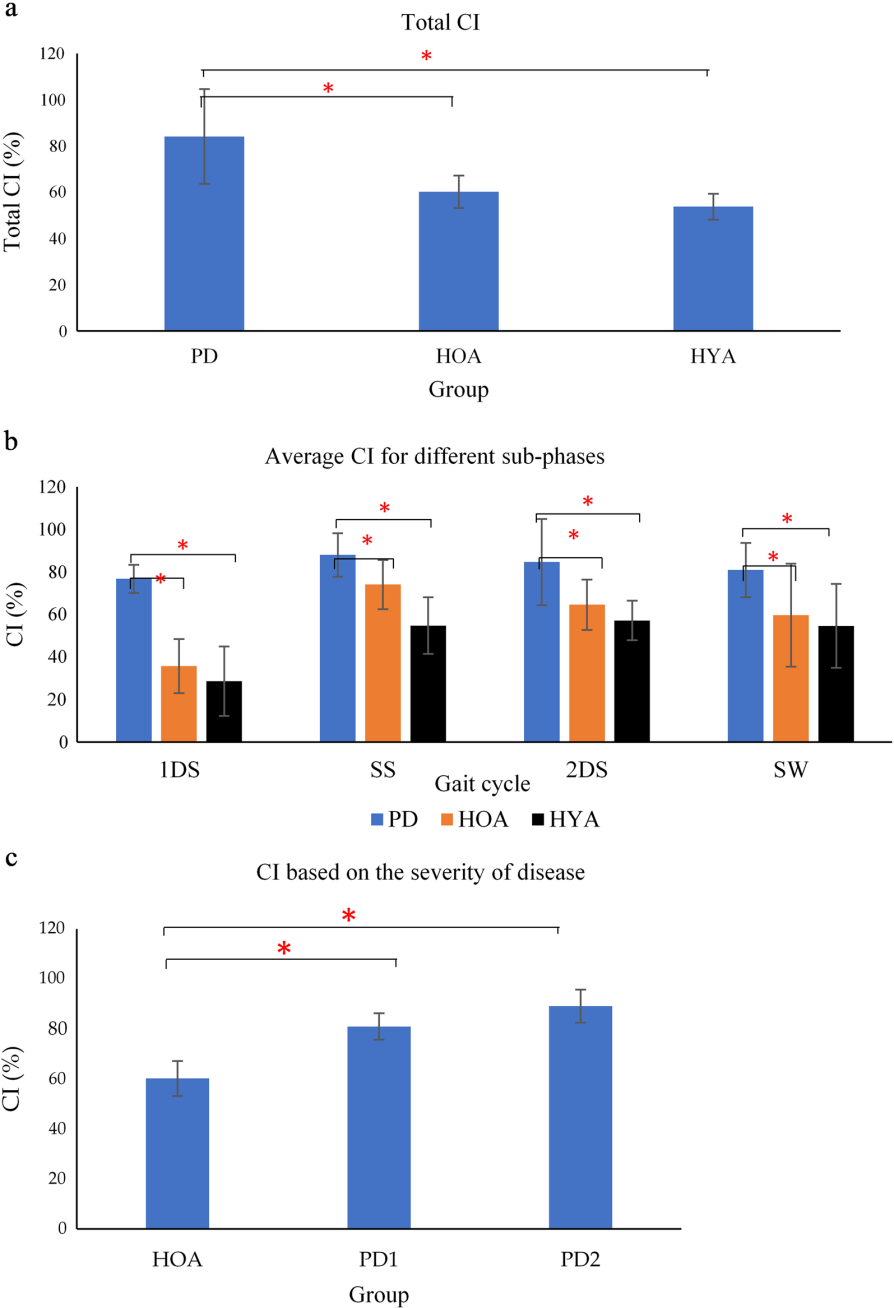
Bar plot showing average CI changes for **a** whole gait cycle, **b** for sub-phases of gait cycle (first double support (1DS), single support (SS), second double support phase (2DS) and swing (SW) phase) for PD, HOA and HYA, **c** based on the severity of disease, (*p (Significance) < 0.05 using Kruskal–Wallis test)

The statistical test showed that there was no significant difference between the two legs and hence the results from only one side have been reported. From Fig. 3a and b, it is seen that the average CI was higher for PD when compared to the control group (HOA and HYA) for a total percentage of gait and during different gait phases—1DS, SS, 2DS and SW. Non-parametric Kruskal–Wallis test shows that there is a statistically significant difference between the three groups—PD, HOA and HYA for total CI (χ^2^(2) = 12.217, *p* = *0.001*), 1DS (χ^2^(2) = 13.698, *p* = *0.000*), SS (χ^2^(2) = 6.383, *p* = *0.047*), 2DS (χ^2^(2) = 8.201, *p* = *0.021*), SW (χ^2^(2) = 8.030, *p* = *0.025*). Bonferroni post-hoc test was carried out between PD and HOA and PD and HYA as shown in Table 3, with a significance of *p* < 0.05. Figure 3c shows that the average CI was higher for different levels of severity—PD1, PD2 when compared to the age-matched control (HOA).

**Table 3:** The table showing the statistical significance (*p*) between PD and HOA, PD and HYA for different EMG features

EMG features	Gait cycle	*p*-value	*p*-value
		PD and HOA	PD and HYA
CI	1DS	< 0.001	< 0.001
	SS	0.042	0.015
	2DS	0.030	0.024
	SW	0.033	0.024
AI-TA muscle	1DS	0.057	0.049
	SS	0.048	0.042
	2DS	0.045	0.045
	SW	0.003	0.002
AI-MG muscle	1DS	0.052	0.049
	SS	0.042	0.042
	2DS	0.048	0.042
	SW	0.03	0.024
MI-TA muscle	1DS	0.030	0.045
	SS	0.042	0.033
	2DS	0.036	0.027
	SW	0.039	0.021
MI-MG muscle	1DS	0.042	0.036
	SS	0.045	0.039
	2DS	0.048	0.033
	SW	0.045	0.035

### EMG modulation of TA and MG muscle

**Fig. 4:**
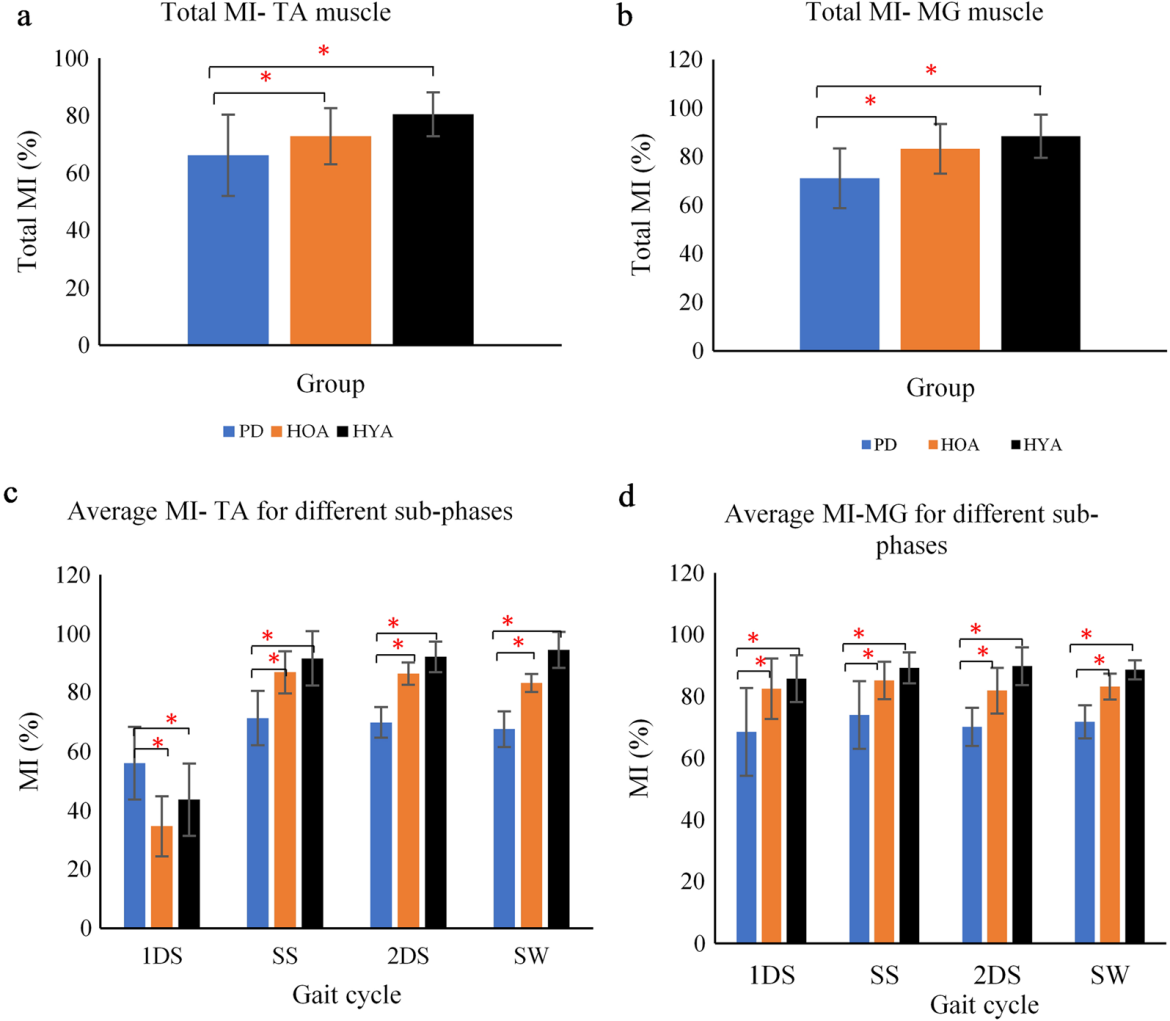
Average MI values of TA and MG muscle for whole gait cycle (**a**, **b**) different sub-phases (first double support (1DS), single support (SS), second double support phase (2DS) and swing (SW) phase) (**c**, **d**) respectively, (*p (Significance) < 0.05 using Kruskal–Wallis test)

Total gait cycle average MI for TA muscle (Fig. 4a) was 66.19 ± 12.3, 72.81 ± 10.21, and 80.45 ± 8.91 for people with PD, HOA, and HYA participants respectively, while for MG muscle (Fig. 4b), this was 71.13 ± 14.3, 83.20 ± 9.81, and 88.37 ± 7.64. Figures 4c and d show the average MI of both TA and MG for the 4 sub-phases of gait. Non-parametric Kruskal–Wallis test shows that there is a statistically significant difference between the three groups-PD, HOA and HYA for total MI-TA muscle (χ^2^(2) = 6.654, *p* = *0.046*), 1DS (χ^2^(2) = 7.187, *p* = *0.038*), SS (χ^2^(2) = 7.012, *p* = *0.039*), 2DS (χ^2^(2) = 7.458, *p* = *0.031*), SW (χ^2^(2) = 7.569, *p* = *0.029*), for total MI-MG muscle (χ^2^(2) = 6.090, *p* = *0.048*), 1DS (χ^2^(2) = 7.011, *p* = *0.039*), SS (χ^2^(2) = 6.776, *p* = *0.042*), 2DS (χ^2^(2) = 6.958, *p* = *0.040*), SW (χ^2^(2) = 6.980, *p* = *0.040*). Bonferroni post-hoc test was carried out between PD and HOA and PD and HYA for both MI-TA and MI-MG muscle as shown in Table 3, all with a significance of *p* < 0.05. MI of people with PD for TA muscle was lower for 3 sub-phases of gait (SS, 2DS, SW) and higher for 1DS. For MG muscle, the MI value was lower for all 4 sub-phases of gait (1DS, SS, 2DS and SW).

### Asymmetric index

**Fig. 5:**
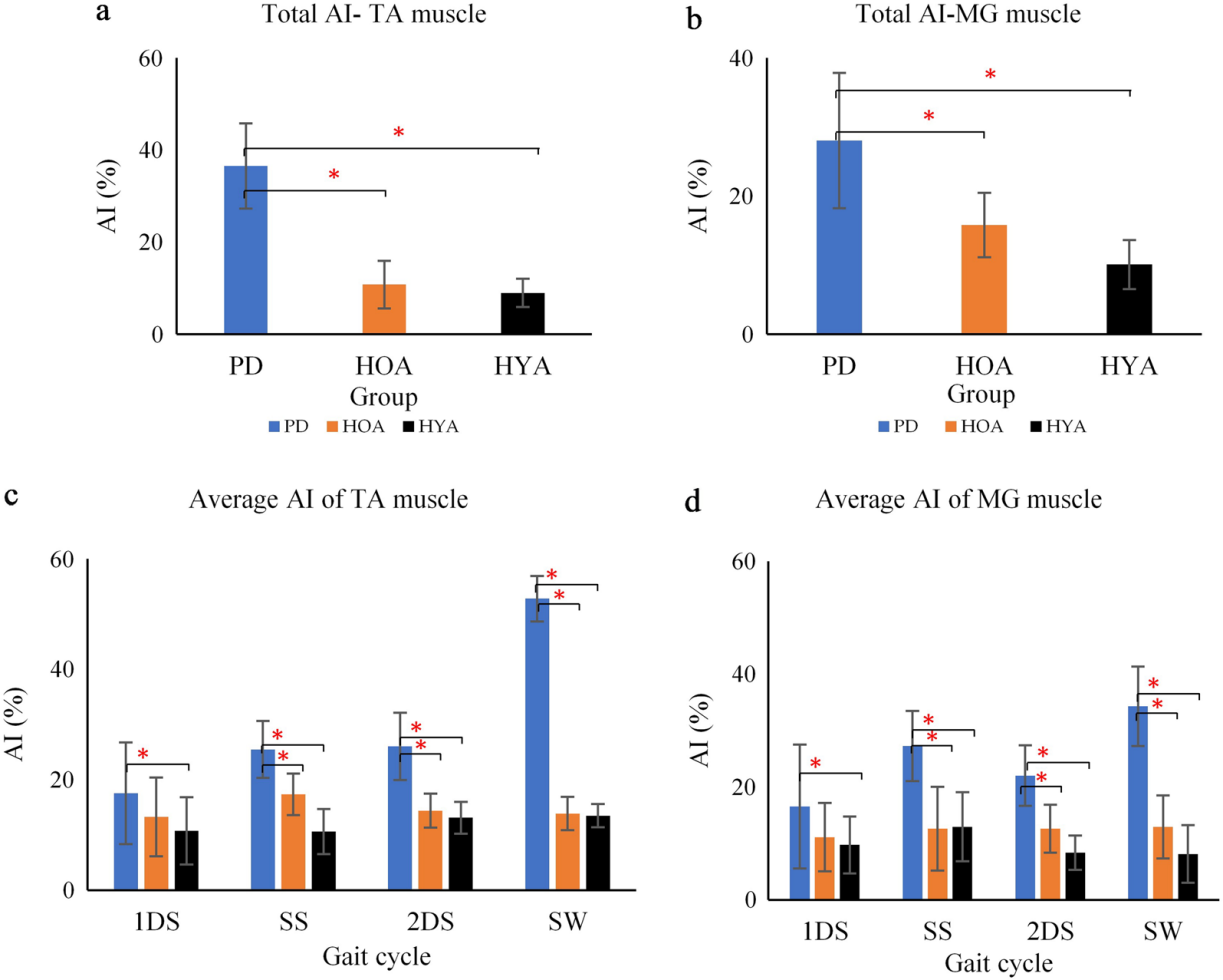
Average AI values of TA and MG muscle for whole gait cycle (**a**, **b**) different sub-phases (first double support (1DS), single support (SS), second double support phase (2DS) and swing (SW) phase) (**c**, **d**) respectively, (*p (Significance) < 0.05 using Kruskal–Wallis test)

Figure 5 shows the average AI values for the full gait cycle (a, b) and for the different sub-phases of gait (c, d) of TA and MG muscle. People with PD had significantly higher average AI for total gait cycle as shown in Fig. 5a and b, and all sub-phases (*p* < 0.05), except during the 1DS, as shown in Fig. 5c and d. People with PD had highest asymmetry during the swing phase of gait. Non-parametric Kruskal–Wallis test shows that there is a statistically significant difference between the three groups—PD, HOA and HYA for total AI-TA muscle (χ^2^(2) = 5.987, *p* = *0.050*), SS (χ^2^(2) = 6.532, *p* = *0.044*), 2DS (χ^2^(2) = 6.459, *p* = *0.045*), SW (χ^2^(2) = 11.235, *p* = *0.002*), for total AI-MG muscle (χ^2^(2) = 6.012, *p* = *0.049*), 1DS (χ^2^(2) = 5.987, *p* = *0.050*), SS (χ^2^(2) = 6.776, *p* = *0.042*), 2DS (χ^2^(2) = 6.459, *p* = *0.045*), SW (χ^2^(2) = 8.078, *p* = *0.026*). Bonferroni post-hoc test was carried out between PD and HOA and PD and HYA for both AI-TA and AI-MG muscle as shown in Table 3, all with a significance of *p* < 0.05. We noticed a higher value of EMG-RMS in left side for PD (17 patients out of 24), HOA (12 out of 24) and HYA (4 out of 24).

Seventeen people with PD (out of 24), 12 HOA (out of 24) and 4 HYA (out of 24) had higher left side activity, even though they were all right side dominant for their lower limb. Table 3 shows the statistical significance value between PD and HOA, and PD and HYA groups for the three sEMG features.

### Correlation study between sEMG features and clinical parameters

Table 4 shows the correlation coefficient and statistical significance between total CI and MI of TA muscle with clinical parameters. CI showed a strong positive and significant correlation with UPDRS bradykinesia (Item 3.14) and UPDRS PIGD, while MI of TA muscle was related to UPDRS rigidity (Item 3.3), and UPDRS PIGD. There was also a moderate significant correlation between UPDRS III and MI of TA muscle, which may be because of other sub-scores of UPDRS III. We have only considered strong positive and significant correlation between MI of TA muscle and UPDRS rigidity (Item 3.3), and UPDRS PIGD. Increase in CI is associated with increase in gait impairment, slowness in movement and postural instability. Increase in MI-TA is associated with increase in rigidity, gait impairment and postural instability. The MI of MG muscle and AI of both TA and MG muscle were not significantly correlated with clinical parameters and hence have not been reported.

**Table 4:** Correlation study of EMG features and clinical features

Clinical variables	Scale range	Total CI	Total MI-TA muscle
UPDRS postural stability (Item 3.12)	0–2	+ 0.620 (0.054)	+ 0.677(0.023)
UPDRS rigidity (Item 3.3)	0–5	+ 0.674 (0.011)	+ 0.810 (0.033)
UPDRS gait (Item 3.10)	0–3	+ 0.759 (0.002)	+ 0.820 (0.002)
UPDRS body bradykinesia (Item 3.14)	0–3	+ 0.778 (0.006)	+ 0.625(0.015)
UPDRS PIGD	0–20	+ 0.858 (0.018)	+ 0.788(0.012)
Year of disease	1–10	+ 0.554 (0.003)	+ 0.687 (0.07)
H & Y scale	1–3	+ 0.347 (0.241)	+ 0.578 (0.061)
UPDRS III	9–41	+ 0.388 (0.157)	+ 0.738 (0.017)

The ρ (*p*-value)—Spearman correlation coefficients (ρ) is indicated with level of significance (*p*). The acronyms used in the table—UPDRS III—Unified Parkinson’s Disease Rating Scale, UPDRS PIGD—UPDRS III Postural Instability and Gait Disturbance, H & Y Scale—Hoehn and Yahr scale

**Fig. 6:**
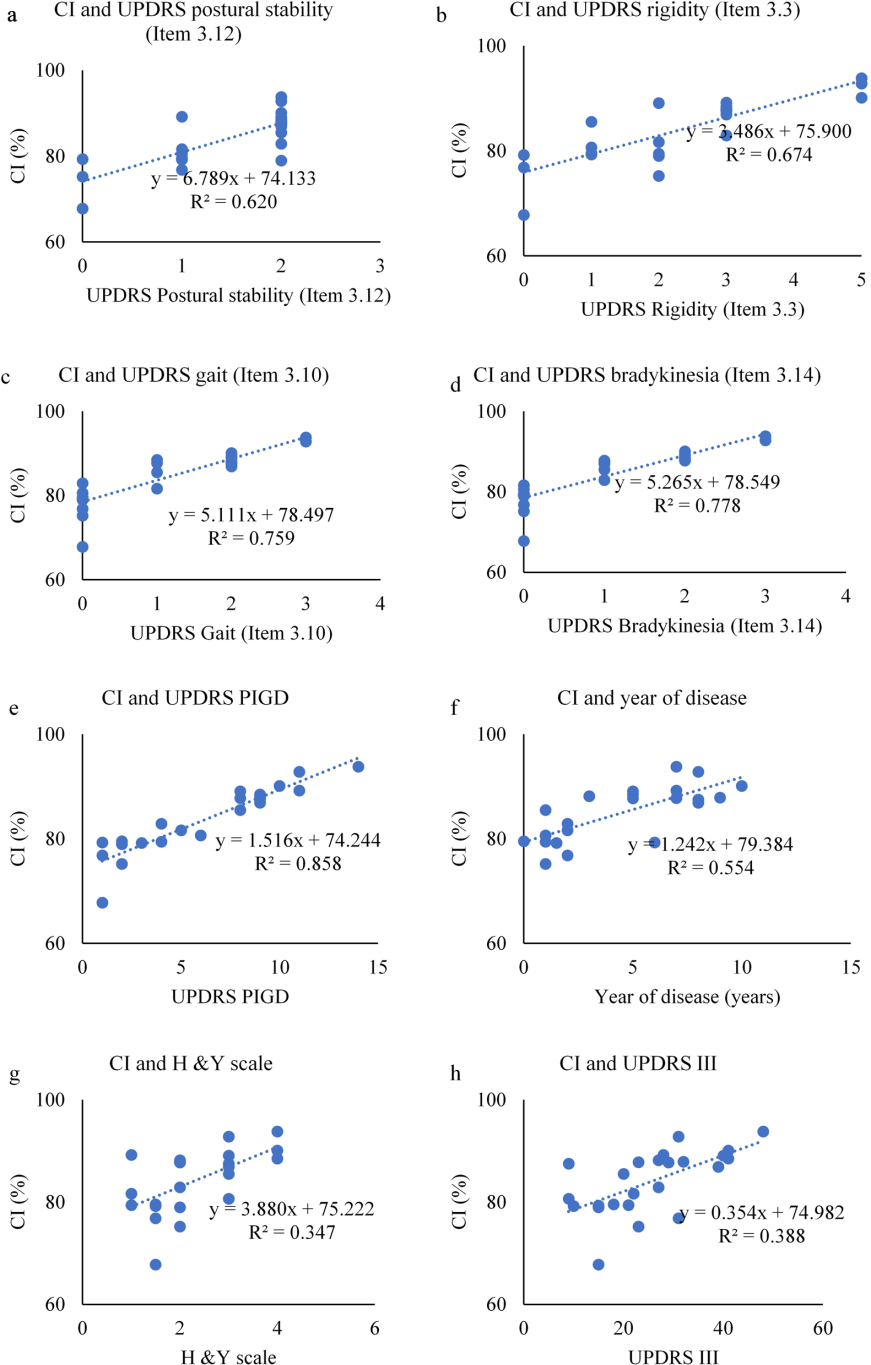
Scatterplot between CI and clinical features—**a** UPDRS postural stability (Item 3.12), **b** UPDRS rigidity (Item 3.3), **c** UPDRS gait (Item 3.10), **d** UPDRS bradykinesia (Item 3.14), **e** UPDRS PIGD, **f** year of disease, **g** H & Y scale, and **h** UPDRS III (The correlation coefficient (R^2^) and the linear fitted regression equation is given))

**Fig. 7:**
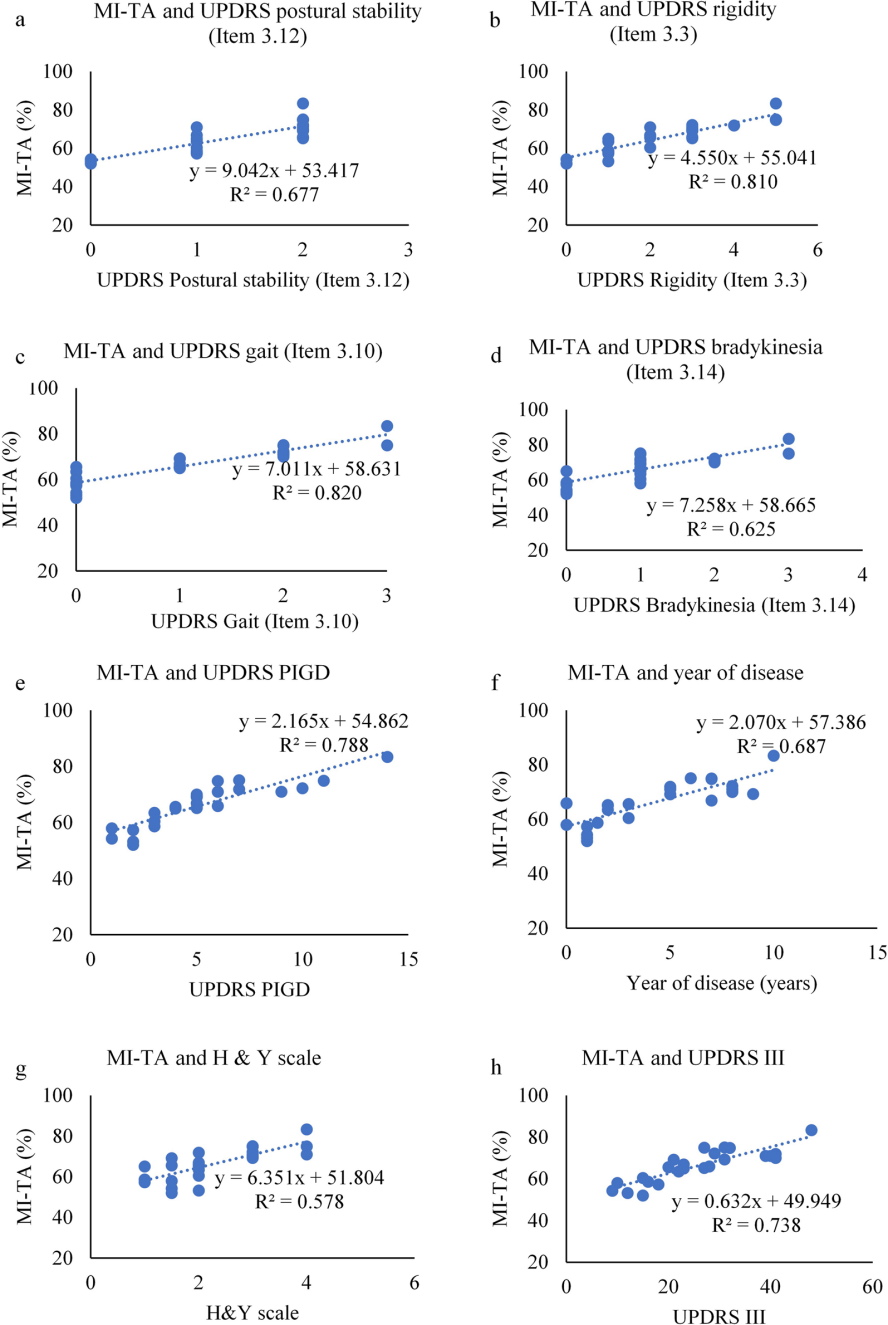
Scatterplot between MI-TA and clinical features—**a** UPDRS postural stability (Item 3.12), **b** UPDRS rigidity (Item 3.3), **c** UPDRS gait (Item 3.10), **d** UPDRS bradykinesia (Item 3.14), **e** UPDRS PIGD, **f** year of disease, **g** H & Y scale, and **h** UPDRS III (The correlation coefficient (R^2^) and the linear fitted regression equation is given))

Figure 6 shows the scatterplot between the CI and the clinical features. Figure 7 shows the scatterplot between the MI-TA values and clinical features. The linear equation and correlation coefficient values for each plot have also been provided.

From Fig. 6, it is observed that CI correlates strongly with UPDRS-PIGD, UPDRS-Bradykinesia, and UPDRS-Gait, while there is moderate correlation with UPDRS-Posture and UPDRS-Rigidity. The correlation between CI and UPDRS-III and H&Y was weak.

From Fig. 7, it is observed that MI of TA strongly correlates with UPDRS-Rigidity, UPDRS-Gait, UPDRS-PIGD and UPDRS-III, while there was moderate correlation with UPDRS-Posture, UPDRS-Bradykinesia, and years of disease.

## Discussion

This study has investigated the differences in the muscle activity of people with PD with low PIGD (average 5.29), age-matched controls and young controls for the different sub-phases of the gait cycle. Relative muscle activity, co-activation index, sEMG modulation and gait asymmetry during sub-phases of gait were compared between groups. The observations are discussed below in four sections.

### Muscle activity profile of TA and MG muscle

In line with the literature, it was visually observed from sEMG graph that the age-matched controls exhibit greater activation of TA during midstance [26] while people with PD have reduced activation of TA during stance [17]. Sub-phase analysis revealed an increased activity of TA during the early and mid-swing phases of the gait, and RMS of MG was less during all gait phases for PD.

Decline in the production of dopamine neurotransmitter leads to the excessive inhibition of the basal ganglia loop, and people with PD have loss of habitual patterns associated with walking and postural control [45]. TA muscle functions as the dorsiflexor of the foot and maintains balance during lateral transfer [46]. The impairment in habitual actions, reduced postural control and limited control of the foot leads to risk of falls [21, 22] which may cause PD being unable to relax the TA.

### Co-activation of TA and MG muscle

Our results are in line with similar literature, which studied when on treadmill, where it was reported that the people with PD exhibit increased co-activation [17] compared to controls, and older controls have higher CI compared to young controls [20]. Increased co-activation is reported as a neuromotor strategy when postural stability is challenged [47].

This study has shown that for all groups, the 1DS had the lowest CI, and it is highest CI during SS, which may be explained in terms of the need for stabilization of the muscles during that phase of gait [47]. CI of controls (irrespective of age) modulated over the cycle, but this was not the case in PD. It was also observed that the CI was significantly higher for all sub-phases of gait for PD, with the greatest increase during the 1DS (*p* < 0.001). This can be interpreted that people with PD, even with low PIGD (5.29) appear to need extra ankle joint support all the time, while controls need that only during the SS phase.

There was a significant difference of CI between HOA and PD1, HOA and PD2 (*p* < 0.05). Excessive co-activation of the ankle muscle may be one of the causes of gait impairment, and maybe the precursor to observable symptoms. Increased co-activation of agonist-antagonist muscles results in an increased rigidity at the ankle and impaired gait in people with PD [17] and increased metabolic cost [48]. This would explain our results which shows a strong correlation of co-activation with bradykinesia and UPDRS-PIGD, which is a simple assessment of balance and gait [49]. The high CI of PD in stage 1 shows that there is the potential of using CI of the TA/MG for early-stage diagnosis.

### sEMG modulation of TA and MG muscle

MI describes the ability to activate and inhibit the muscle as required for the movement [22]. The MI of sub-phases of the gait reveals that for people with PD, MI for TA muscle was lower in the 1DS phase, while MI for MG muscle was lower during all sub-phases. The reduced ability to regulate the muscle activation may be linked to the impairment in the proprioceptive system [50], resulting in poor modulation of muscle [51].

Another finding was that the MI-TA muscle strongly correlated with the clinical features—rigidity, gait and UPDRS-PIGD score. Lack of modulation of the muscle activity may be an indicator of rigidity symptoms of PD and cause reduced work efficiency. However, short length of the walk could be a compounding factor as it may cause cognitive loading, which could also contribute towards reduced efficient management of muscle activity [52].

### Gait lateral asymmetry of TA and MG muscle

People with early-stage PD have higher gait asymmetry [28], and increased AI [53] compared with HOA. Ours is the first study where the sub-phases of gait have been investigated.

As the first step, our results confirm that AI value of people with PD is higher than HOA [53]. It also shows that control participants AI is approximately the same for all the sub-phases, while for people with PD, AI value was significantly higher during the SW phase for TA muscle and during SS and SW phase for MG muscle. PD symptoms are not bilateral, and a bias towards the non-dominant side [54] has been reported. People with PD have increased “left hemisphere susceptibility,” in the left nigrostriatal pathway, which is more affected than the right, irrespective of handedness [55], commonly seen in early stage of PD. This may have caused lower muscle activity in the right lower extremity. The unsupported phase of gait of people with PD is highly asymmetrical. Increased asymmetry and reduced modulation of muscle could be due to the reduced ability of basal ganglia to generate repetitive and habitual movements [53], or other factors such as modified spinal cord pathways.

## Conclusion

The study has shown the importance of monitoring the sub-phases of gait of people with early-stage PD to monitor them for gait impairment. It has found a significantly higher co-activation of the TA and MG muscles, reduced modulation and increased asymmetry in early people with PD compared with age-matched controls and young controls. They have reduced muscle activity, ability to inhibit antagonist, and modulate their muscle activities, which is most evident during the gait initiation phase.

While many people with PD had low posture and gait difficulty scores (5.29 + 3.07/20), the difference in the sEMG between PD and HOA was significant. This was for both, people with stage 1 and stage 2 PD, indicating the potential of its use for early detection of gait impairment and subsequent monitoring of the progression for the disease. This uses wearable sensors and has the advantage over observing the gait parameter which may be affected by number of other confounding factors. Another advantage of this method is that this can be investigated during a level, straight-line short distance walking inside an office using wearable sensors. This has the potential for early detection of gait impairment among people with PD and prevent falls.

## Limitation of the study

This is a cross-sectional study and does not give direct evidence of its applications for detecting or monitoring early symptoms of the disease. There are three more limitations of the present study: (i) short walking distance and thus small number of gait cycles, (ii) only PD-ON state patients were tested, and (iii) postural instability was only assessed using UPDRS-PIGD score. The short walking distance may be insufficient to investigate some of the aspects of PD gait and may also contribute towards cognitive loading. Medication can also significantly affect the tonic state of muscle, where the difference may be even greater in the OFF state of medication. Lastly, the severity of the gait impairment was measured using UPDRS-PIGD scores which are simple clinical assessment measure for postural instability.

## Data Availability

The de-identified data is available on the University database.
